# Evaluation of DNA interaction, genotoxicity and oxidative stress induced by iron oxide nanoparticles both *in vitro* and *in vivo*: attenuation by thymoquinone

**DOI:** 10.1038/s41598-019-43188-5

**Published:** 2019-05-06

**Authors:** Mohd Owais Ansari, Nuzhat Parveen, Md Fahim Ahmad, Ab Latif Wani, Shumaila Afrin, Yusra Rahman, Sana Jameel, Yasir Akhtar Khan, Hifzur R. Siddique, Mohammad Tabish, G. G. H. A. Shadab

**Affiliations:** 1 Cytogenetics and Molecular Toxicology Lab, Section of Genetics, Department of Zoology, Aligarh Muslim University, Aligarh, 202002 Uttar Pradesh India; 2 Molecular Cancer Genetics & Translational Research Lab, Section of Genetics, Department of Zoology, Aligarh Muslim University, Aligarh, 202002 Uttar Pradesh India; 3 Department of Biochemistry, Faculty of Life Sciences, Aligarh Muslim University, Aligarh, 202002 Uttar Pradesh India; 4 Department of Zoology, Aligarh Muslim University, Aligarh, 202002 Uttar Pradesh India

**Keywords:** Cytogenetics, Biochemistry, Biomarkers

## Abstract

Iron oxide nanoparticles (IONPs) are known to induce cytotoxicity in various cancer cell lines through the generation of reactive oxygen species (ROS). However, the studies on its potential to induce toxicity in normal cell lines and *in vivo* system are limited and ambiguity still exists. Additionally, small molecules are known to interact with the DNA and cause damage to the DNA. The present study is designed to evaluate the potential interaction of IONPs with DNA along with their other toxicological effects and subsequent attenuation by thymoquinone both *in vitro* (primary lymphocytes) and *in vivo* (Wistar rats). IONPs were characterized by TEM, SEM-EDS, and XRD. The results from DNA interaction studies showed that IONPs formed a complex with DNA and also got intercalated between the base pairs of the DNA. The decrease in percent cell viability of rat’s lymphocytes was observed along with an increase in ROS generation in a dose-dependent manner (50, 100, 200, 400 and 800 μg/ml of IONPs). The genetic damage in *in vivo* might be due to the generation of ROS as depletion in anti-enzymatic activity was observed along with an increase in lipid peroxidation in a dose–dependent manner (25, 50, 100 mg/kg of IONPs). Interestingly, supplementation of thymoquinone in combination with IONPs has significantly (*P* < 0.05) attenuated the genetic and oxidative damage in a dose-dependent manner both *in vitro* and *in vivo*. It can be concluded that thymoquinone has the potential to attenuate the oxidative stress and genetic toxicity *in vitro* and *in vivo*.

## Introduction

The extensive use of magnetic nanoparticles in the biomedical field increases its potential threat to human health^1^. Iron oxide nanoparticles (IONPs) have been extensively used in biomedical sciences such as magnetic hyperthermia, magnetic resonance imaging, gene delivery, tissue imaging etc.^1–6^. Besides these applications, extensive use of IONPs in cosmetics and textile industries also leads to their accumulation that possesses a threat to human health along with other living organisms^7^. Nanoparticles are defined as the engineered particles with the size range less than 100 nm^5,6^. However, within a few decades, IONPs had made progress for its application for effective cancer therapy. Several preliminary studies on various cell lines have made a promise to kill cancerous cells effectively following treatment with IONPs in a dose-dependent manner^8–10^. Similarly, the exposure of copper ferrite (CuFe_2_O_4_) nanoparticles to human breast cancer (MCF-7) cells and Cobalt iron oxide (CoFe_2_O_4_) nanoparticles to human liver cells (HepG2) also induced cytotoxicity and apoptosis via ROS generation^11,12^. The general mechanism behind the cytotoxicity of IONPs against cancerous cells was due to the induction of oxidative stress through the generation of Reactive Oxygen Species (ROS)^13–15^. However, the studies on the impacts of IONPs on human health have not been properly addressed. A few studies have shown that IONPs have potential to induce toxicity via ROS generation in both dose and time-dependent manner^16–18^. Sundarraj *et al*.^19^ also reported that IONPs have potential to cause toxicity in reproductive organs such as seminal vesicle and prostate gland via induction of oxidative stress markers and alteration to heat shock gene expression^19^.

Natural antioxidants are active components of herbal extracts that neutralize the generation of ROS before it causes any damage to tissue or macromolecules^20^. *Nigella sativa* and its components such as thymoquinone (TQ), carvacrol, t-anethole, and 4-terpineol are also known for many years as a natural antioxidant^21^. However, TQ, the most bioactive component of *Nigella sativa* has been studied for its antioxidant property^22^.

The interaction of small molecules with nucleic acids has been extensively studied as it helps in the development of a better understanding at the molecular level and also contributes to the development of a rational drug design system^23^. Small molecules interact with DNA, broadly through two mechanisms, intercalation and groove binding, although electrostatic forces also contribute to the interaction of charged molecules with DNA^24^. Since intercalating agents are known to cause DNA damage, it becomes imperative to study the mode of interaction of nanoparticles (NPs) with DNA^25^.

In the present study, we investigated the potential of IONPs induced oxidative and genetic damage and their interaction with DNA. The study was also aimed to optimize the level of TQ for its anti-oxidative property both *in vitro* and *in vivo*. The DNA binding potential of IONPs was evaluated by UV-vis spectroscopy and dye displacement assay. IONPs induced *in vitro* cytotoxicity in rat’s primary lymphocytes was measured by MTT assay, and ROS was estimated. IONPs induced oxidative and genetic damage was also investigated in *in vivo* system by cytogenetic analysis, comet assay, and biochemical assays.

## Materials and Methods

### Chemicals

Iron (II, III) oxide (Fe_3_O_4_) nanoparticles (CAS Number: 1317-61-9), Thymoquinone (CAS Number: 274666), Calf thymus DNA (ct-DNA), Acridine Orange (AO), 4′,6-diamidino-2-phenylindole (DAPI), and Hoechst were procured from Sigma Aldrich, India. Cell culture medium RPMI-1640 and Fetal Bovine Serum (FBS) were procured from Gibco, India. The antibiotic-antimycotic solution was procured from Thermo Scientific India. Cyclophosphamide (CP) and Colchicine (CAS 64-86-8) were procured from Merck, India. All other chemicals used were of analytical grade purity.

### Characterization of Iron Oxide Nanoparticles

IONPs were characterized for size, morphology, and shape by Transmission Electron Microscopy (TEM), Scanning Electron Microscopy (SEM) coupled with Energy Dispersive X-ray Spectroscopy (EDS), and X-ray Diffraction (XRD). For TEM and SEM-EDS, IONPs were suspended in phosphate buffer saline (PBS, pH = 7.4) and were ultrasonicated prior to coating on the grid. TEM and SEM (Joel, JEM 2100, Japan) coupled with EDS were used for characterization. For XRD, Miniflex, Desktop X-ray diffractometer (Rigaku, Japan) was used.

### Interaction Studies between IONPs and DNA

#### UV-Vis Spectroscopy

UV-Vis experiments were performed on a Shimadzu UV 1800 spectrophotometer using quartz cuvette of path length 1 cm. UV-Vis spectrum of a fixed concentration of ct-DNA (50 μM) and DNA-NP complex in increasing concentrations (0–3 mM) was measured in the wavelength range of 200–350 nm.

### Competitive Dye Displacement Assays

Dye displacement assays were carried out using Shimadzu RF 6000 Spectrofluorometer. Acridine orange, DAPI, and Hoechst were used as displacement dyes.

Acridine orange displacement assay was performed using 5 μM each of ct-DNA and AO and titrated with increasing concentrations of NP (0–1.4 mM). Excitation was performed at 490 nm and emission was recorded from 500–700 nm.

Similarly, for Hoechst and DAPI, 5 μM of Hoechst/DAPI and ct-DNA were titrated with increasing concentrations of NP. Excitation wavelength used in the case of Hoechst was 343 nm and emission was recorded between 375 and 600 nm. The excitation and emission wavelength used in case of DAPI were 358 nm and from 400–600 nm respectively. Slit widths were fixed at 3 nm.

### In Vitro Studies

#### Lymphocytes Isolation

The rat was sacrificed and the spleen was taken out and was minced in phosphate buffer saline (PBS). The homogenate was then passed through 70 µm strainer in PBS. The obtained cells were centrifuged at 1200 rpm for 10 min. The supernatant was discarded, and the pellet was resuspended in red blood cell lysis buffer (BD Pharmingen) and tapped gently for 10 min. After that, the cell suspension was raised to avoid further lysis. The cell suspension was then again centrifuged at 1200 rpm for 10 min. The obtained pellet was raised to the desired volume of RPMI-1640 (Gibco) supplemented with 0.5% antibiotic-antimycotic solution and 10% FBS. Cell viability was checked by Trypan Blue Dye Exclusion Αssay. The cell with >97% viability was taken into consideration for culture experiments.

### MTT Assay

The cytotoxic effect of IONPs and its amelioration by thymoquinone was assessed by MTT assay^26^. Approximately 1 × 10^6^/well in 200 μl culture media in RPMI-1640 media (Gibco) containing 10% FBS (Gibco), 0.5% antibiotic-antimycotic solution (Thermo Fisher) were seeded in flat bottom 96 well plate. For cytotoxicity and its inhibition by thymoquinone, the cells were treated with the different concentrations (50 μg/ml, 100 μg/ml, 200 μg/ml, 400 μg/ml and 800 μg/ml) of INOPs in absence and in presence of different concentrations (25 μM, 50 μM, 75 μM, and 100 μM) of thymoquinone, respectively. After 24 h of incubation at 37 °C with 5% CO_2_ supply in a humidified incubator, 5 μl of MTT solution from 5 mg/ml stock solution (prepared in PBS) was added and further incubated for 3.5 h under same conditions. After that, the pellet was centrifuged for 10 min at 2200 rpm to settle down the floating cells. Finally, the media was discarded gently, and 100 μl of DMSO was added to dissolve formazan crystal. The dissolved crystals gave a purple color. The plate was read at 572 nm wavelength on ELISA plate reader. The percent cell viability was calculated as per the formula: mean OD of vehicle control/ mean OD of treated sample x100.

### Measurement of Reactive Oxygen Species (ROS)

The level of ROS in lymphocytes *in vitro* conditions was estimated as described earlier with slight modifications^16^. Briefly, 1 × 10^6^/well were treated with different concentrations of IONPs (50 μg/ml, 100 μg/ml, 200 μg/ml, 400 μg/ml and 800 μg/ml) in the absence and in the presence of different concentrations of (25 μM, 50 μM, 75 μM and 100 μM) of thymoquinone for 24 h. At the end of the experiment, the cells were centrifuged for 10 min at 2200 rpm. The cells were washed two times with PBS and centrifuged similarly. The washed cells were incubated in 100 μl of 100 μM of DCFH-DA solution at 37 °C for 30 min. After that, the fluorescent intensity was measured at 485/520 nm (excitation/emission) wavelength with ELISA plate reader. The data were plotted as percent increase in fluorescence intensity.

### In Vivo Studies

#### Animals and Experimental Design

Male Wistar rats (120–150 gm) were used for the experiment. Animals were housed in a well-maintained animal house (room temperature 18–25 °C; relative humidity 45–55%, 12/12 h light/dark cycle). Animal diet and water were given *ad libitum* and rats were acclimatized to the laboratory conditions before the experimental setup. The experiments were carried out under the strict guidance and principles of the institutional animal ethical committee and guidelines accredited by the Committee for the Purpose of Control and Supervision of Experiments on Animals (CPCSEA). The study was approved by the Committee for the Advance Studies and Research (CASR). The experimental protocol was approved by Institutional Animal Ethics Committee, Aligarh Muslim University, Aligarh, India.

Animals were divided into 9 groups with 6 animals in each group. The route of administration was intraperitoneal (i.p.). Nanoparticles were suspended in phosphate buffer saline (PBS, pH = 7.4) and were sonicated for 20 min before administration to the animals once for 7 consecutive days.

Group 1: Negative control and received only PBS.

Group 2: Positive control and received Cyclophosphamide (CP, 40 mg/kg b.w.).

Group 3: Treated with IONPs (25 mg/kg b.w.).

Group 4: Treated with IONPs (50 mg/kg b.w.).

Group 5: Treated with IONPs (100 mg/kg b.w.).

Group 6: Treated with TQ (6 mg/kg b.w.) along with IONPs (100 mg/kg b.w.).

Group 7: Treated with TQ (12 mg/kg b.w.) along with IONPs (100 mg/kg b.w.).

Group 8: Treated with TQ (18 mg/kg b.w.) along with IONPs (100 mg/kg b.w.).

Group 9: Treated with TQ (24 mg/kg b.w.) along with IONPs (100 mg/kg b.w.).

### Chromosomal Aberration (CA) Assay

The clastogenic potential of IONPs and its amelioration by thymoquinone were evaluated by chromosomal aberration assay. The metaphase chromosomes were prepared according to Benoniet *et al*.^27^ with required modification by Parveen *et al*.^28^. For metaphase chromosome preparation, the animals were treated with colchicine (2 mg/kg) 2 h before sacrificing the animals. Following metaphase cell arrest, animals were sacrificed, and femurs were dissected out. Next, with KCl (0.075 M), the bone marrow was flushed out in centrifuge tubes with the help of an insulin syringe (by gentle agitation). The flushed-out bone marrow cells were resuspended in KCl (hypotonic treatment) and left in a water bath (37 °C) for 20 min. After hypotonic treatment, cells were centrifuged at 300 g for 10 min, and the obtained supernatant was discarded. The remaining pellets were resuspended in a little amount of KCl and then fixed in freshly prepared chilled fixative (methanol: acetic acid in the ratio of 3:1) for 20 min, and preferably overnight. The fixed cells were centrifuged, and the steps were repeated 2–3 times for proper washing. Again, the supernatants were discarded, and the remaining pellets were resuspended in fixative. The suspended cells were then made to fall from a considerable height onto a pre-chilled clean slide tilted at a 45° angle. The slides were then left to air dry. The prepared slides were then stained with 5% Giemsa stain for 10 min and again left to air dry. Slides were then observed for metaphase chromosomes under a light microscope (Nikon, Japan) with 100X oil immersion lens for different chromosomal aberrations. Scoring of fifty well-spread metaphase plates was done per slide.

### Micronucleus Test (MNT)

Micronucleus test was performed to evaluate the IONPs induced clastogenic effect and its amelioration by thymoquinone in rat bone marrow cells. The MN test was done as per the protocol of Schmidt^29^ and modified by Ahmad *et al*.^30^. Briefly, after completion of the duration of treatment, the femur bones of the rats were dissected out. The bone marrow was flushed out with fetal bovine serum (FBS) and was centrifuged at 300 g for 10 min and the supernatant was discarded. The pellets were resuspended in a little amount of FBS. The smears were drawn on the pre-coded clean slides and left to air dry. Differential staining was done in May-Gruenwald stain followed by Giemsa stain (5%). The slides were then mounted in DPX and analyzed under a light microscope (Nikon, Japan). The frequencies of micronucleated polychromatic erythrocytes (MNPCEs) were calculated by scoring 2000 polychromatic erythrocytes (PCEs) per animal.

### Comet Assay

After 24 h of the last treatment, rats were sacrificed, and blood was collected immediately by cardiac puncture in a heparinized blood vial. Lymphocytes were isolated using histopaque in 1:1 and centrifuged at 800 g for 20 min. The buffy layer lymphocytes were taken out and mixed with PBS in 1:1 and again centrifuged at 250 g for 10 min. The supernatant was discarded, and the cell pellets were diluted in PBS (1:1) to form a suspension. The isolated lymphocytes were mixed with low melting point agarose (LMPA, 0.8%) and were placed on frosted slides coated with 1% normal melting point agarose (NMPA), covered with a coverslip to make uniform layer and was placed on ice packs for 10 min to solidify. A third layer was also made with 0.5% of LMPA. The prepared slides were then subjected to lysis by keeping them in lysis buffer for 2 h. After lysis, slides were then immersed in the alkaline buffer in the electrophoretic tank for 20 min for DNA unwinding followed by electrophoresis for 30 min. After electrophoresis, the slides were dipped in neutralization buffer for 5 min (2–3 times). Finally, the slides were stained with ethidium bromide (EtBr, 20 μg/ ml) and analyzed under a fluorescent microscope. Comet tail lengths of 50 nuclei were analyzed, and the tail length was calculated using image analysis software (Komet 5.5) attached to an Olympus (CX41) fluorescent microscope (Olympus) and a COHU 4910 integrated CC camera equipped with 510–560 nm excitation and 590 nm barrier filters (COHU). Migration of DNA from the nucleus i.e. the tail length was measured for lymphocyte DNA damage.

### Biochemical Assays

Biochemical assays were performed for the evaluation of oxidative stress markers against IONPs induced toxicity and its amelioration by thymoquinone. Briefly, liver tissues were taken out and homogenized in PBS (pH = 7.4) in the ratio of 1:10 in a glass homogenizer. The homogenate was centrifuged, and the supernatant was kept for different biochemical assays by spectrophotometer.

### Catalase (CAT) Activity

The CAT activity was evaluated according to the protocol of Aebi^31^. Briefly, 3 ml of the reaction mixture was prepared by mixing 1.9 ml of 0.05 M phosphate buffer (pH = 7.4), 100 μl of homogenate and 1 ml of 30 mM H_2_O_2._ The absorbance was recorded at an interval of 30 sec for 3 min at a UV-wavelength of 240 nm. The molar extinction coefficient of H_2_O_2_ (436 mol l^−1^ cm^−1^) was used to calculate the CAT enzyme activity, and the values were expressed in U/mg protein

### Superoxide Dismutase (SOD) Activity

The SOD activity was measured as per the protocol of Marklund and Marklund on the principle of its ability to inhibit the auto-oxidation of pyrogallol^32^. Briefly, 50 μl of homogenate was added to 2.85 ml of Tris succinate buffer (0.05 M, pH = 8.2). The reaction was started by adding 100 μl of 8.0 mM pyrogallol. The change in absorbance was recorded at an interval of 30 sec for 3 min at vis-wavelength of 420 nm. For reference, a reaction mixture was used that contained 50 μl of distilled water instead of homolysate. The SOD activity was expressed as U/mg protein.

### Glutathione (GSH) Estimation

GSH level was estimated as per the protocol given by Jollow *et al*.^33^. Briefly, 500 μl of 4% sulphosalicylic acid was added to 100 μl of homogenate and was incubated for 1 h at 4 °C. Then the mixture was centrifuged at 1200 g for 15 min, and the supernatant was taken out. The reaction was started by mixing supernatant with 2.2 ml of phosphate buffer (0.1 mM, pH = 7.4) and 0.4 ml of DTNB (4 mg/ml). The absorbance of the yellow color developed was recorded at vis-wavelength of 412 nm. The concentration of GSH was expressed as nmol/ mg protein.

### Lipid Peroxidation (LPO) Assay

LPO was determined as per the protocol of Beuge and Aust^34^. Briefly, 1 ml of homogenate was mixed with 2 ml of TBA-trichloro acetic acid-hydrochloric acid (TCA-HCL), followed by heating in a boiling water bath for 20 min. Then, the mixture was cooled at room temperature and centrifuged at 400 g for 10 min. The supernatant was taken, and its absorbance was measured spectrophotometrically at 532 nm against a blank (which contains all the reagents except supernatant). The level of MDA formation was calculated by using the molar extinction coefficient of MDA (1.56 M^−1^ cm^−1^), and the obtained values were expressed in nmol/mg protein

### Protein Estimation

Total protein was measured by the total protein kit from Ark Ray Health Care, India, following the manufacturer’s guidelines.

### Statistical Analysis

All the data have been expressed as Mean ± SD. One-way ANOVA was done to test the level of significance. GraphPad Prism 7.0 was used to analyze the data. The statistical significance level was set at *p* < 0.05.

## Results

### Characterization of Nanoparticles

**Figure 1:**
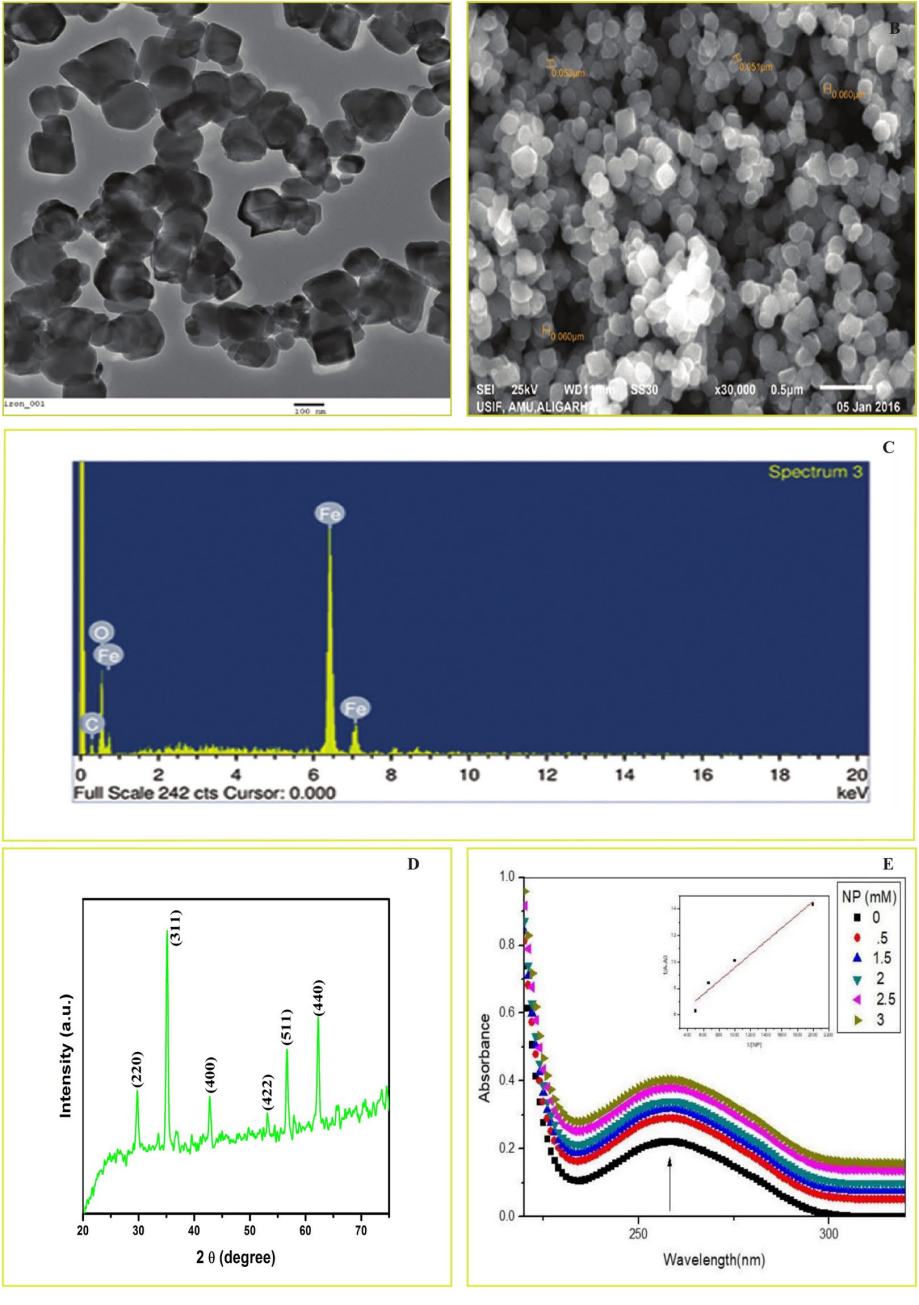
Characterization of IONPs. Transmission Electron Microscopy and Scanning Electron Microscopy showed the shape and size of the IONPs (**A**,**B**), Energy Dispersive X-ray Spectroscopy showed that Fe and O_2_ are the major elements present in the powder (**C**), X-ray Diffraction showed that IONPs are in crystalline form (**D**). UV spectra of ct-DNA in the absence and presence of increasing concentrations of nanoparticles (0–3 mM). The inset shows a plot of 1/ΔA vs. 1/[NP] (**E**).

The TEM and SEM results showed that the Fe_3_O_4_ NPs are spherical in shape and the average size of the nanoparticle was approximate ~60 nm (Fig. 1A,B). The EDS analysis result showed that the Fe and O_2_ are the primary elements present in the powder (Fig. 1C). The XRD data also showed that the Fe_3_O_4_ nanoparticles are crystalline in nature and the diffraction peaks 220, 311, 400, 422, 511, and 440 confirm that nanoparticles consist of Fe_3_O_4_ (Fig. 1D)_._

### DNA Interaction Studies

#### UV-Vis Spectroscopy Analysis

UV-Vis spectroscopy is a useful tool to detect alterations in the secondary structure of biomolecules such as DNA after its interaction with small molecules. The absorption spectra of DNA were recorded in the absence and presence of IONPs with increasing concentration. A concentration depended hyperchromism was observed with no appreciable shift in the absorption maximum of DNA, indicating complex formation between IONPs and DNA (Fig. 1E).

The value of dissociation constant was determined using the equation:

$${\rm{1}}/{\rm{\Delta }}{\rm{A}}={{\rm{K}}}_{{\rm{d}}}/({{\rm{\Delta }}{\rm{A}}}_{\infty }[{\rm{S}}])+{\rm{1}}/{{\rm{\Delta }}{\rm{A}}}_{\infty }$$

where ∆A = A − A0 and A0 and A represent absorbances of DNA in the absence and presence of IONPs [S] represents the concentration of IONPs, and A_∞_ represents the change in absorbance on ligand saturation.

From a plot of 1/∆A vs. 1/[S], the value of K_d_ was calculated and was used to calculate the value of association constant using the relation

$${{\rm{K}}}_{{\rm{d}}}={\rm{1}}/{{\rm{K}}}_{{\rm{a}}}$$

The value K_a_ was determined to be 1 × 10^3^ M^−1^.

### Competitive Displacement Assay

Using fluorescence spectroscopy, DNA binding dyes acridine orange (AO), Hoechst and DAPI were used to decipher the binding mode of NPs to DNA. AO is a known intercalator whereas DAPI and Hoechst bind in the groove of DNA. If a drug intercalates between base pairs of the DNA molecule, it will displace the intercalating dye and result in fluorescence quenching. However, if the drug binds within the grooves of DNA, no significant fluorescence quenching will be observed using an intercalating dye. It will, however, displace a groove binding dye and result in fluorescence quenching. Table 1 shows the comparative evaluation of fluorescence quenching constants Ksv (Stern-Volmer quenching constant) in the presence of AO, Hoechst, and DAPI. Since the Ksv for AO displacement assay was much greater than DAPI and Hoechst displacement assays, it can be inferred that IONPs intercalates within the base pairs of DNA.

**Table 1:** Quenching constants for dye displacement assays.

Dye	Ksv (×10^3^ M^−1^)	R^2^
Acridine orange	2.02	0.999
Hoechst 33258	0.05	0.961
DAPI	0.04	0.538

Ksv: quenching constant, R^2^: Chi-square.

### In Vitro Studies

#### Cytotoxicity Assessment of IONPs In Vitro and its Attenuation by Thymoquinone

**Figure 2:**
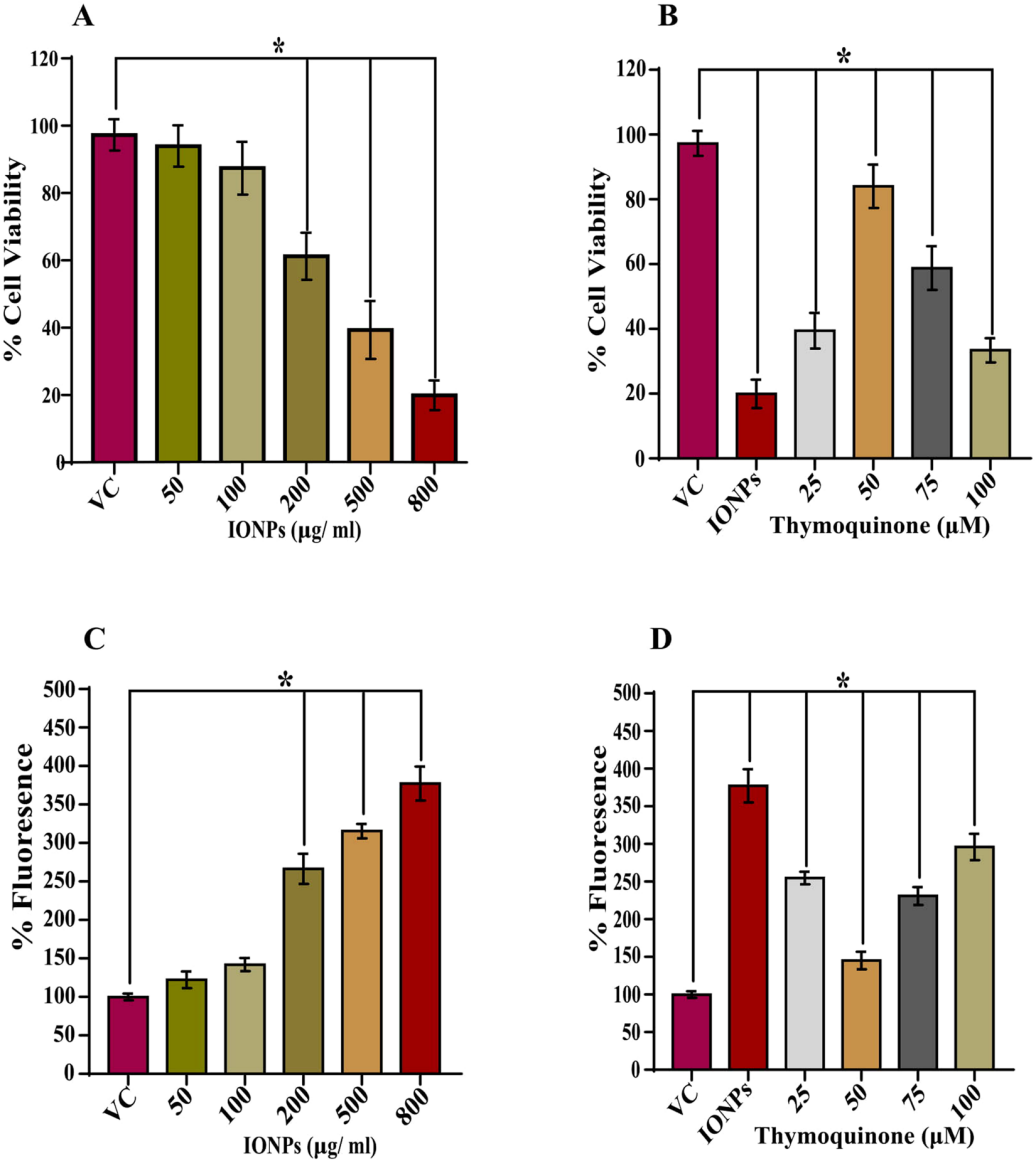
IONPs induced cytotoxicity *in vitro*. Bar diagrams are showing a dose-dependent decrease in % cell viability in lymphocytes and its attenuation and optimization by thymoquinone (**A**,**B**). A dose-dependent increase in ROS generation and its attenuation and optimization by thymoquinone (**C**,**D**). Data are statistically significant at (*p < 0.05). VC, *Vehicle Control*; PC, *Positive Control*.

The IONPs showed dose-dependent cytotoxicity in lymphocytes after 24 h of treatment. At a lower dose (50 and 100 μg/ml of IONPs), the decrease in cell viability was insignificant as compared to control. However, there was significant (*p* < 0.05) decrease in cell viability of lymphocytes at 200 μg/ml of IONPs (61.26% vs. control) while at 800 μg/ml of IONPs, 19.96% decrease in cell viability of lymphocytes was observed (Fig. 2A).

Interestingly, after 24 h of TQ (25, 50, 75, and 100 μM) treatment along with IONPs (800 μg/ml), a significant (*p* < 0.05) increase in cell viability was observed. However, the maximum increase in cell viability was observed at 50 μM. The result also showed that at higher dose of TQ (75 and 100 μM), there was again a decrease in % cell viability (Fig. 2B).

### ROS Generation by IONPs In Vitro and its Attenuation by Thymoquinone

The ROS generation was estimated by measuring the % increase in fluorescence after IONPs treatment. At a lower dose (50 and 100 μg/ml of IONPs), there was no significant increase in % fluorescence as compared to control. However, the % fluorescence was found to increase significantly (*p* < 0.05) with the increasing doses of all the concentrations (200, 500, and 800 μg/ml) of IONPs (Fig. 2C).

After 24 h of TQ (25, 50, 75, and 100 μM) treatment along with IONPs (800 μg/ml), the % fluorescence decreased significantly (p < 0.05) up to 50 μM of TQ. However, at higher doses of TQ (75, and 100 μM), again a significant increase in % fluorescence was observed (Fig. 2D).

### In Vivo Studies

#### Chromosomal Aberrations and its attenuation by Thymoquinone

**Figure 3:**
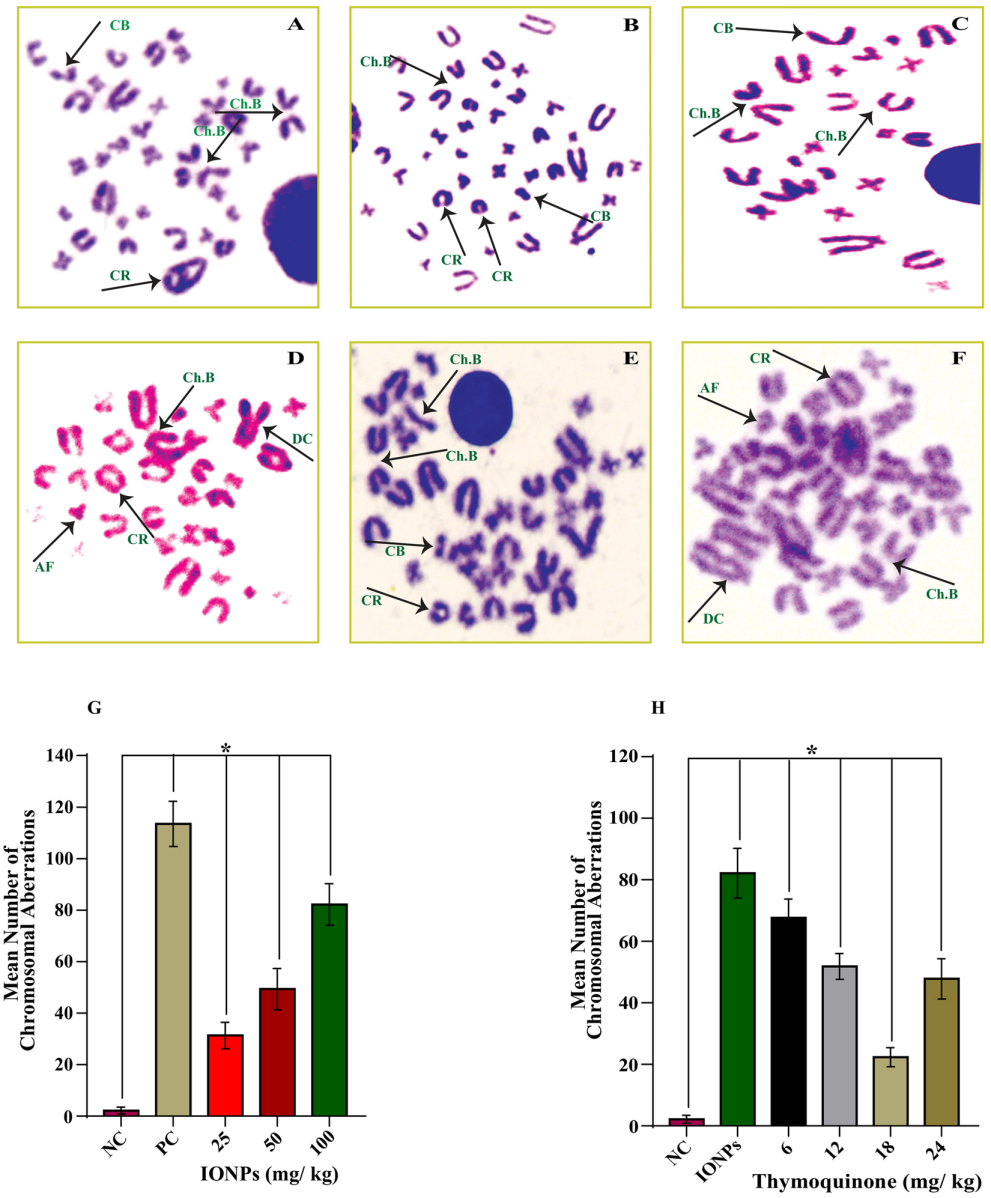
IONPs induced Chromosomal aberration in bone marrow cells of the animals treated for one week. Photographs showing different types of structural chromosomal aberrations such as chromosome breaks, chromatid breaks, and chromosomal ring (**A**), chromosome breaks, chromatid breaks and chromosomal ring (**B**), chromosome breaks, and chromatid breaks (**C**), chromosome breaks, ring formation, dicentric chromosome, and acentric chromosome (**D**), chromosome breaks, chromatid breaks and ring formation (**E**), Dicentric chromosome, acentric chromosome, and chromosome breaks (**F**). Graph showing IONPs induced chromosomal aberrations in bone marrow cells of rat (**G**) and its attenuation and optimization by thymoquinone. (**H**) Data are statistically significant at (*p < 0.05). *CB*, *Chromatid Breaks*; *Ch*.*B*, *Chromosome Breaks*; *CR*, *Chromosomal Ring*; *AF*, *Acentric Fragments*; *DC*, *Dicentric Chromosome*; NC, *Negative Control*; PC, *Positive Control*.

IONPs treatment for one week showed different types of chromosomal aberrations such as chromosome breaks, chromatid breaks, acentric fragments, dicentric chromosomes, and chromosomal rings. However, chromosome and chromatid breaks were found with the highest frequency and dicentric chromosomes with the lowest frequency (Fig. 3A–F). The mean values of chromosomal aberrations were found to be 02.16 ± 1.32 (negative control), 113.50 ± 8.82 (positive control), 31.33 ± 5.16 (IONPs, 25 mg/kg), 49.33 ± 8.06 (IONPs, 50 mg/kg), and 82.16 ± 8.08 (IONPs, 100 mg/kg) respectively. The obtained results showed that IONPs induced a statistically significant (*p* < 0.05) increase in the mean values of chromosomal aberrations in a dose-dependent manner versus negative control (Fig. 3G).

However, the co-treatment of TQ (6, 12, 18, and 24 mg/kg) along with IONPs (100 mg/kg) showed a significant (*p* < 0.05) decrease in the mean values of chromosomal aberrations versus positive control in a dose-dependent manner (Fig. 3H). The mean values of chromosomal aberrations were found to be 67.66 ± 6.12, 51.83 ± 4.1 TQ, 31.66 ± 5.27 TQ, and 47.83 ± 6.55 at 6 mg/kg, 12 mg/kg, 18 mg/kg, and 24 mg/kg of TQ respectively. It was also observed that at 24 mg/kg of TQ, there was again an increase in the mean number of chromosomal aberrations as compared to 18 mg/kg of TQ (Fig. 3H).

### Micronucleus Induction and its attenuation by Thymoquinone

**Figure 4:**
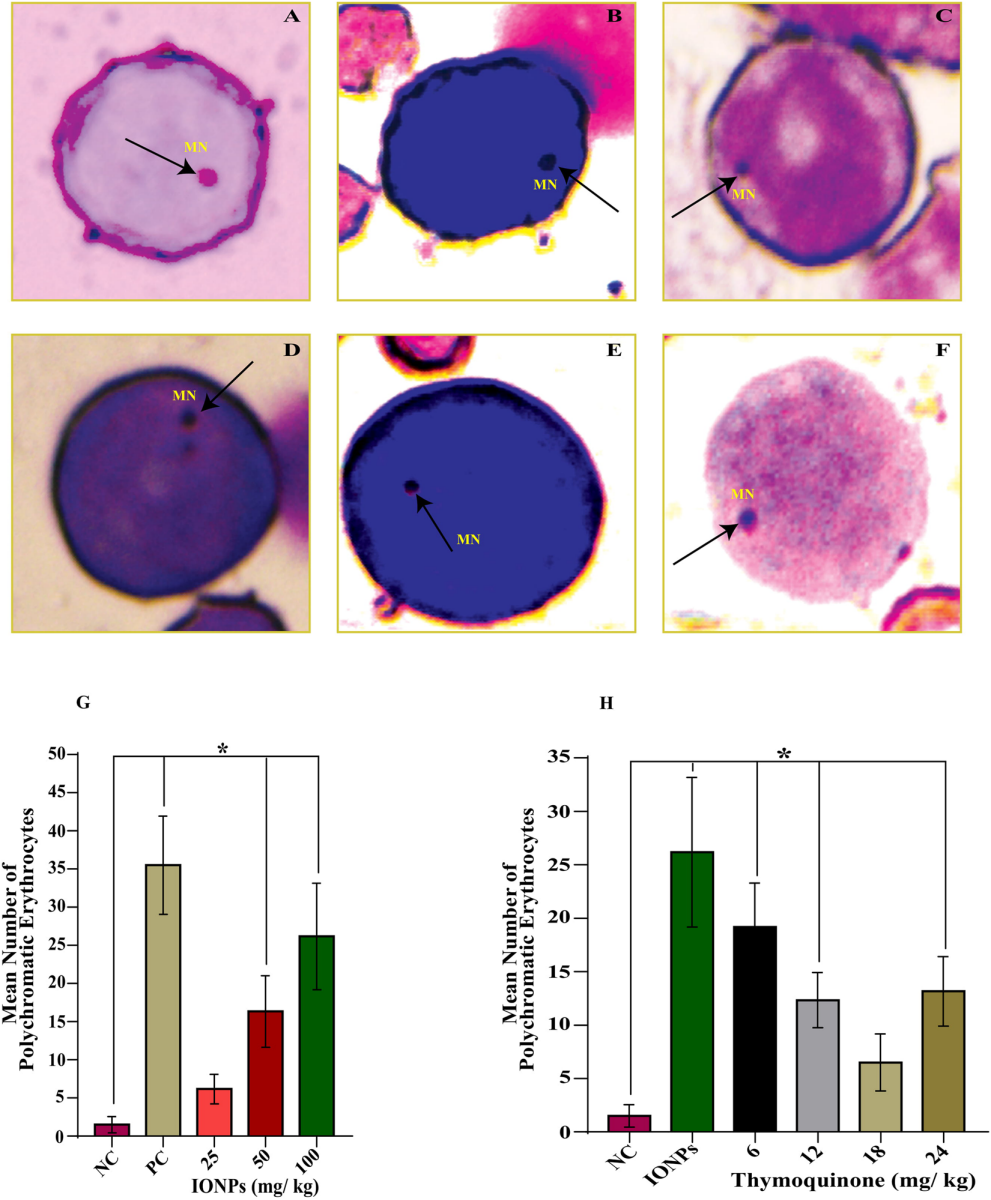
IONPs induced micronucleus formation in bone marrow cells of the animals treated for one week. Photographs showing micronucleus formation in polychromatic erythrocytes (**A**–**F**). Graph showing IONPs induced micronucleus formation in bone marrow cells of rat (**G**) and its attenuation and optimization by thymoquinone. (**H**) Data are statistically significant at (*p < 0.05). *MN*, *Micronucleus*; NC, *Negative Control*; PC, *Positive Control*.

The daily dose of IONPs for one week showed induction of Micronucleated Polychromatic Erythrocytes (MNPCEs) formation in a dose-dependent manner (Fig. 4A–F). The mean number of MNPCEs were found to be 1.50 ± 1.04 (negative control), 35.50 ± 6.44 (positive control), 6.16 ± 1.94 (IONPs, 25 mg/kg), 16.33 ± 4.67 (IONPs, 50 mg/kg), and 26.16 ± 6.99 (IONPs, 100 mg/kg) respectively. The obtained results showed that IONPs induced a statistically significant (*p* < 0.05) increase in mean number of MNPCEs formation in a dose-dependent manner versus negative control (Fig. 4G).

Attenuation of micronucleus formation with TQ was also observed. The simultaneous treatment of IONPs (100 mg/kg) along with TQ, showed a significant (*p* < 0.05) decrease in mean number of MNPCEs versus positive control in a dose-dependent manner (Fig. 4H). The mean numbers of MNPCEs were found to be 19.16 ± 4.11, 12.33 ± 2.58, 06.50 ± 2.66, and 13.16 ± 3.25 at 6 mg/kg, 12 mg/kg, 18 mg/kg, and 24 mg/kg of TQ respectively. It was also observed that at 24 mg/kg of TQ, there was again an increase in mean number of MNPCEs as compared to 18 mg/kg of TQ (Fig. 4H).

### Comet Assay

**Figure 5:**
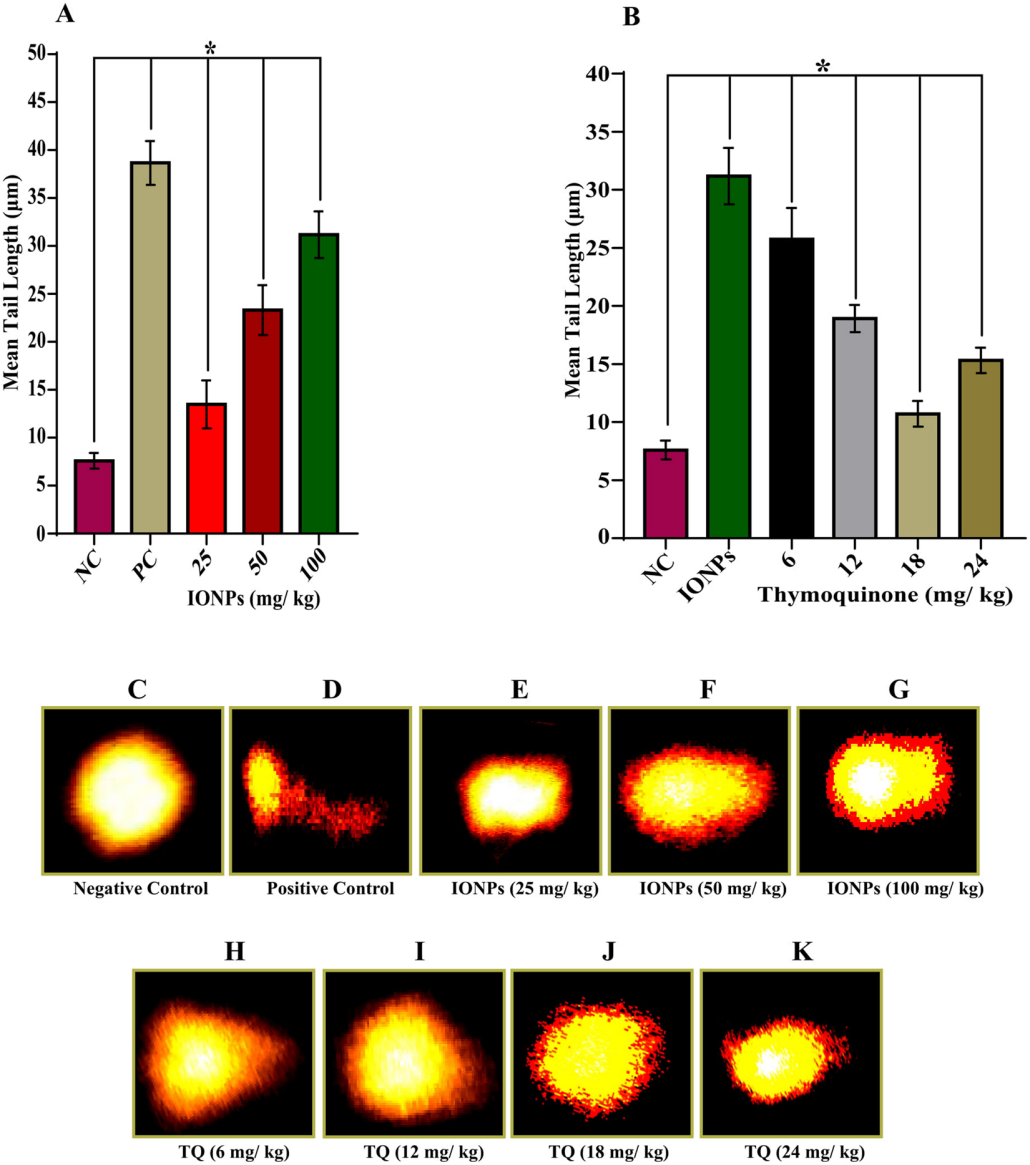
IONPs induced DNA damage in blood cells of animals treated for one week and its attenuation by thymoquinone. Bar diagrams showing an increase in mean tail length in a dose-dependent manner after IONPs exposure (**A**) and its attenuation and optimization by thymoquinone (**B**), photographs showing an increase in mean tail length in a dose-dependent manner after IONPs exposure (**C**–**G**), and its attenuation by co-exposure of thymoquinone (**H**–**K**). Data are statistically significant at (*p < 0.05). NC, *Negative Control*; PC, *Positive Control*; TQ, *Thymoquinone*.

After one week of IONPs exposure, the mean tail length was found to be 7.59 ± 0.81 (negative control), 38.67 ± 2.27 (positive control), 13.48 ± 2.49, 23.31 ± 2.59 and 31.18 ± 2.42 (25, 50 and 100 mg/kg of IONPs) respectively. There was an increase in mean tail length with the increased concentration of IONPs as compared to control. The obtained result showed a significant (*p* < 0.05) increase in mean tail length versus negative control in a dose-dependent manner (Fig. 5A,C–G).

However, the co-exposure of TQ along with IONPs ameliorates the extent of DNA damage induced by IONPs (100 mg/kg). The mean tail length was found to be 25.75 ± 2.67, 18.91 ± 1.17, 10.71 ± 1.11, and 15.31 ± 1.09 for 6, 12, 18, and 24 mg/kg of TQ respectively. The obtained result showed that the TQ significantly (*p* < 0.05) attenuates the extent of DNA damage caused by IONPs (Fig. 5B,H–K).

### Biochemical Analysis

#### Catalase (CAT) Activity

**Figure 6:**
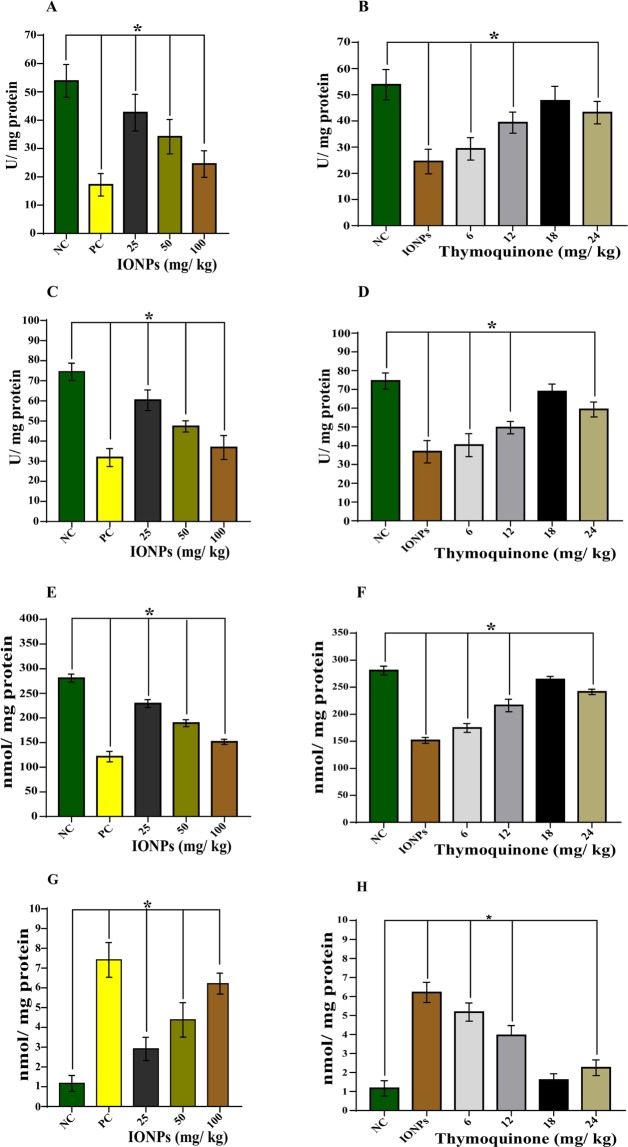
IONPs induced oxidative stress in the animals treated for one week and its attenuation by thymoquinone. Bar diagrams showing decrease in catalase activity after IONPs exposure and its attenuation by co-exposure of thymoquinone (**A**,**B**), decrease in superoxide dismutase activity after IONPs exposure and its attenuation by co-exposure of thymoquinone (**C**,**D**), decrease in reduced glutathione level after IONPs exposure and its attenuation by co-exposure of thymoquinone (**E**,**F**), an increase in malondialdehyde (MDA) level after IONPs exposure and its attenuation by co-exposure of thymoquinone (**G**,**H**). Data are statistically significant at (*p < 0.05). NC, *Negative Control*; PC, *Positive Control*.

After exposure to IONPs for one week, the obtained catalase activity was found to be 53.83 ± 5.81 (negative control), 17.16 ± 3.97 (positive control), 42.66 ± 6.47 (IONPs, 25 mg/kg), 34.16 ± 6.080 (IONPs, 50 mg/kg), and 24.50 ± 4.72 (IONPs, 100 mg/kg) U/mg protein. The observed result showed that with the increasing doses of IONPs, there was statistically significant (*p* < 0.05) decrease in catalase activity (Fig. 6A).

The co-exposure of TQ along with IONPs showed that the organism replenished the activity of catalase in a dose-dependent manner. The catalase activity after co-exposure of TQ (6, 12, 18, and 24 mg/kg) along with IONPs (100 mg/kg) was found to be 29.33 ± 4.27, 39.33 ± 4.03, 47.66 ± 5.53, and 43.16 ± 4.30 U/mg protein respectively. The result showed a statistically significant (*p* < 0.05) increase in catalase activity in a dose-dependent manner after TQ co-exposure, however, at 18 mg/kg of TQ, maximum optimization was observed (Fig. 6B).

### Superoxide Dismutase (SOD) Activity

After one week of exposure of IONPs, a dose-dependent depletion in the activity of SOD was observed. The obtained results were found to be 74.50 ± 4.32, 34.16 ± 3.27, 60.33 ± 5.16, 44.50 ± 2.88, and 34.50 ± 4.63 U/mg protein for negative control, positive control, and 25, 50, and 100 mg/kg of IONPs respectively. The result showed that with the increasing doses of IONPs, there was statistically significant (*p* < 0.05) decrease in SOD activity (Fig. 6C).

The co-exposure of TQ regulates the imbalance in SOD activity caused by IONPs treatment. The results obtained after TQ co-exposure along with IONPs (100 mg/kg) were 40.33 ± 6.12, 49.66 ± 3.26, 68.83 ± 4.07, and 59.33 ± 3.98 U/mg protein for 6, 12, 18, and 24 mg/kg of TQ respectively. The result showed a statistically significant (*p* < 0.05) elevation in the SOD activity followed by TQ treatment in a dose-dependent manner. However, the maximum optimization was observed at 18 mg/kg of TQ (Fig. 6D).

### Glutathione (GSH) level Estimation

The results showed that there was a dose-dependent depletion in GSH level after IONPs treatment to the animal for one week. The obtained results were found to be 280.66 ± 8.21, 121.66 ± 10.53, 229.16 ± 8.03, 189.5 ± 7.09, and 151.66 ± 5.31 U/mg protein for negative control, positive control, and 25, 50, and 100 mg/kg of IONPs respectively. The result showed that with the increasing doses of IONPs, there was statistically significant (*p* < 0.05) decrease in GSH level (Fig. 6E).

The co-exposure of TQ along with IONPs helps the organisms to replenish the GSH level. The result was found to be 174.50 ± 8.21, 216.16 ± 11.51, 264.16 ± 5.91, and 241.33 ± 5.01 U/mg protein for 6, 12, 18, and 24 mg/kg of TQ against 100 mg/kg of IONPs respectively. The observed result showed a statistically significant (*p* < 0.05) elevation in the GSH level followed by TQ treatment in a dose-dependent manner. However, the maximum optimization was observed at 18 mg/kg of TQ (Fig. 6F).

### Lipid Peroxidation (LPO) Assay

The treatment of IONPs to the animal for one week showed an elevated level of MDA adduct formation in a dose-dependent manner. The obtained result was found to be 1.16 ± 0.40, 7.41 ± 0.87, 2.91 ± 0.58, 4.38 ± 0.86, and 6.21 ± 0.53 nmol/mg protein for negative control, positive control, and 25, 50, 100 mg/kg of IONPs respectively. The result showed that with the increasing doses of IONPs, there was statistically significant (*p* < 0.05) increase in MDA level (Fig. 6G).

The co-exposure of TQ along with IONPs showed its protective role by reducing the extent of MDA production. The obtained result was found to be 5.18 ± 0.47, 3.95 ± 0.52, 1.60 ± 0.33, and 2.25 ± 0.40 nmol/mg protein for 6, 12, 18, and 24 mg/kg of TQ against 100 mg/kg of IONPs respectively. The result showed that with the increasing doses of IONPs, there was statistically significant (*p* < 0.05) reduction in MDA level. However, the maximum optimization was observed at 18 mg/kg of TQ (Fig. 6H).

## Discussion

In recent times, the toxic effects of nanoparticles have raised serious concern over its application in various fields of science. Iron is an important constituent of hemoglobin, several enzymes and electron transport system of the cells^35^. However, above the threshold level, iron also contributes to the production of ROS that causes oxidative stress and induces damage to the tissues and organs of the body^28^. IONPs induced oxidative stress through ROS generation, and oxidative damage to both *in vitro* and *in vivo* systems was evaluated earlier also, but the studies on its amelioration with natural antioxidants are yet to be studied precisely^﻿36﻿^.

Interestingly, we have evaluated the IONPs interaction with DNA and observed that IONPs have the potential to get intercalated between the DNA base pairs in a concentration-dependent manner. The finding suggests that IONPs may cause DNA damage directly by intercalating within the base pairs of DNA.

Several *in vitro* studies reported that IONPs induced cellular cytotoxicity via free radical generation such as ROS in various cancer cell lines^8,9,36^. However, the direct application of IONPs and its exposure to human beings have raised concern for its evaluation of cytotoxicity in normal cells. Moreover, reports on its cytotoxicity in normal cells are limited. Cochran *et al*.^37^ reported that IONPs have potential to cause injury to the cells of vascular endothelium through the generation of ROS^37^. Similarly, Gaharwar *et al*.^16^ have also reported that magnetic IONPs cause cellular cytotoxicity to rat lymphocytes via ROS generation in a dose and time-dependent manner^16^. It was also reported that IONPs induced oxidative stress and decreased cell viability of human hepatocytes and human lymphocytes^13,38^. Our study also reported that the exposure of IONPs to rat lymphocytes reduced cell viability in a dose-dependent manner after 24 h, and at the highest concentration (800 μg/ml), the cell viability decreased up to 3 folds. Similarly, in correlation to ROS generation, there was an increase in percent fluorescence after 24 h of IONPs exposure. However, the simultaneous treatment of TQ along with IONPs increased the cell viability in a dose-dependent manner, and above 50 μM, TQ showed its pro-oxidant property.

The micronucleus formation, induction of chromosomal aberrations and extent of DNA damage is a measure of genotoxicity against any toxic chemical^28^. The previous studies related to toxicity of IONPs have created a lot of ambiguities^39^. An increase in micronucleus frequency was observed in human peripheral blood after IONPs exposure in a dose-dependent manner^38^. Similarly, oxidative stress-induced DNA damage was also observed after an intraperitoneal administration of IONPs in mice^18,40^. However, few studies also reported no genotoxicity induced by IONPs^41,42^. In our study, we found that IONPs exposure to Wistar rats increases the frequency of both chromosomal aberration and micronucleus induction along with increased DNA damage in a dose-dependent manner. However, co-exposure of TQ along with IONPs reduces the genotoxic potential of IONPs in a dose-dependent manner. This may be due to the ROS scavenging property of TQ and recovery of the antioxidant system of the animal. However, above a threshold level (18 mg/kg) TQ started behaving like a pro-oxidant and again caused an imbalance to the antioxidant system of the animal with an increase in oxidative and genetic damage^28^.

The exposure of toxic chemical may lead to the production of reactive oxygen species (ROS) which may interfere with the antioxidant system of the organism’s body^43^. The imbalance in redox potential might be a cause of genetic toxicity of IONPs. Several studies have reported that administration of IONPs in rodents induces oxidative stress via generation of ROS that disturbed the antioxidant system of the organism’s body^18,40^. Our results also showed that administration of IONPs for one week causes a decrease in antioxidant enzymes levels such as CAT and SOD along with reduced GSH level. However, the co-exposure of TQ replenished the anti-oxidant system of the animal and caused the recovery of antioxidant enzymes in a dose-dependent manner. The imbalance to anti-oxidant enzyme level is also the cause of lipid peroxidation^44^. Lipid peroxidation causes deterioration of cell membrane and makes it susceptible to further oxidation by free radical^44,45^. In our study, we also observed IONPs induced increased lipid peroxidation in the form of MDA adduct in a dose-dependent manner. However, the co-exposure of TQ significantly reduces the level of MDA adduct formation and thus prevents the loss of cellular integrity.

## Conclusion

The study concludes that IONPs induce genotoxicity in the form of chromosomal aberrations, micronucleus formation, and DNA damage. The DNA damage may be caused by either directly interacting with DNA or through oxidative stress induced generation of ROS. However, the co-exposure of TQ attenuates both oxidative stress and genetic damage induced by IONPs in a dose-dependent manner. Based on our findings, we conclude that TQ can be used as a supplement to avoid any adverse effects of IONPs. However, still more insight is needed from pre-clinical and clinical studies for its validation as a potent supplement against IONPs. The present data also suggest that more care should be taken for the safe use of IONPs and its application in various fields of science and technology.
